# A Case of Cryptococcal Meningitis and Fungemia With Relapse in an HIV-Negative, Non-transplant Patient on Azathioprine Therapy for Mixed Connective Tissue Disorder

**DOI:** 10.7759/cureus.24356

**Published:** 2022-04-21

**Authors:** Ridwan Faruq, Lucia Plichtova, Namita Bhagat, Zane Saul

**Affiliations:** 1 Internal Medicine, Yale New Haven Bridgeport Hospital, Bridgeport, USA; 2 Radiology, Yale New Haven Bridgeport Hospital, Bridgeport, USA; 3 Infectious Disease, Yale New Haven Bridgeport Hospital, Bridgeport, USA

**Keywords:** hydrocephalus, relapse, azathioprine treatment, complicated meningitis, cryptococcus neoformans (c. neoformans)

## Abstract

Cryptococcal meningitis typically occurs in immunocompromised patients. Approximately 80% of cryptococcal infections occur in HIV patients. Non-HIV, non-transplant recipient patients are the least numerous population groups affected by cryptococcal infections. While this group includes patients on biologics and corticosteroids, very few cases have been reported in patients on azathioprine. Cryptococcal meningitis requires antifungal therapy, the duration of which varies among different population groups. Inadequate duration of antibiotics among these groups is one of the most common reasons for relapse; therefore, it is crucial to consider patient demographic when determining antifungal duration.

Here, we report a 68-year-old male with a history of mixed connective tissue disease on azathioprine for six years, who was admitted to the hospital with worsening lethargy. Several days into admission, the patient developed low-grade fevers. Subsequent blood cultures grew *Cryptococcus neoformans*. He was started on liposomal amphotericin B. Lumbar puncture (LP) was done, which demonstrated positive cryptococcal antigen, and flucytosine was added to the treatment regimen. Repeat CSF culture demonstrated no fungal organisms. Amphotericin B was discontinued after 20 days of therapy. Following clinical improvement, he was subsequently discharged on oral fluconazole. One week following discharge, the patient was readmitted with worsening fevers and altered mental status. CSF studies demonstrated the growth of *Cryptococcus* on culture. Liposomal amphotericin B was reinitiated, and fluconazole was continued. Imaging showed hydrocephalus, which worsened despite ventriculoperitoneal shunt. The patient expired following transition to comfort care. In conclusion, cryptococcal meningitis should be considered as a differential in non-HIV, non-transplant patients on azathioprine presenting with fever and worsening lethargy, and 4-6 weeks of induction therapy is required in this patient group to prevent relapse.

## Introduction

Invasive cryptococcosis is a group of fungal infections typically occurring in the setting of immunocompromised states, most commonly presenting as cryptococcal meningitis. The majority of cases occur in patients with cell-mediated immunity deficits, with HIV infection underlying the majority of cases. In the USA, approximately 80% of cryptococcal infections occur in patients with HIV [1]. However, given the advancements in antiretroviral therapy, non-HIV cryptococcosis is becoming increasingly more prevalent, particularly in high-income countries [2,3]. Interestingly, of the infections occurring in the non-HIV group, the proportion of cryptococcal meningitis appears to be lower than in the HIV counterpart [4]. Out of the non-HIV population, the subgroup that is most affected and discussed in the literature is that of solid organ transplant recipients. Non-HIV, non-transplant recipient patients are the least numerous and the least studied of population groups affected by cryptococcal infections. While cryptococcosis is associated with the use of monoclonal antibodies and corticosteroids, very few cases have been reported in patients with azathioprine use. Cryptococcal meningitis requires antifungal therapy, the duration of which varies among different population groups [5]. Inadequate duration of antibiotics among these groups is one of the most common reasons for relapse; therefore, it is crucial to consider patient demographic when determining antifungal duration.

Here, we report the case of a 68-year-old HIV-negative, non-transplant recipient patient on azathioprine therapy presenting with cryptococcal meningoencephalitis and fungemia who was later readmitted due to relapse of infection, which was attributed to the inadequate antimicrobial duration for the patient demographic.

## Case presentation

**Figure 1:**
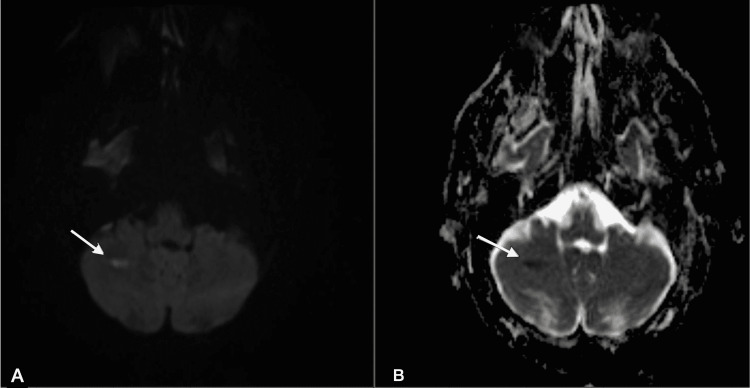
Diffusion-weighted MRI (A) and ADC map (B) shows focus of diffusion restriction with corresponding hypointensity on ADC map (white arrows), suggestive of acute lacunar infarct in the right cerebellar hemisphere.

The patient is a 68-year-old male with a history of hypertension, chronic kidney disease, vasculitis of unclear etiology, and mixed connective tissue disease, who was admitted to the hospital with progressively worsening generalized weakness. He has been on long-term azathioprine therapy for the past six years for mixed connective tissue disease. Initial vitals showed a body temperature of 97.9°F, blood pressure of 148/70 mmHg, and heart rate of 98 bpm. Physical examination was significant for generalized weakness and oral thrush but was otherwise unremarkable. Initial laboratory results were significant for sodium of 126 mmol/L, creatinine of 1.4 mg/dL, and hemoglobin of 10.9 g/dL. Several days into admission, the patient developed low-grade fevers and increasing lethargy. Subsequent blood cultures grew *Cryptococcus neoformans*. He was started on liposomal amphotericin B. Lumbar puncture (LP) was done, which demonstrated positive cryptococcal antigen, and flucytosine was added to the treatment regimen. Subsequent CSF culture grew *Cryptococcus neoformans*. HIV test returned negative. Azathioprine therapy was discontinued. Repeat lumbar puncture two weeks into admission demonstrated persistently elevated cryptococcal antigen titers of 1:2560, and although the patient’s mental status was improving, he remained febrile up to 103°F and persistently encephalopathic. Subsequently, a brain MRI was done to evaluate for cryptococcomas but instead demonstrated acute embolic infarction in the right cerebellum (Figure 1).

**Figure 2:**
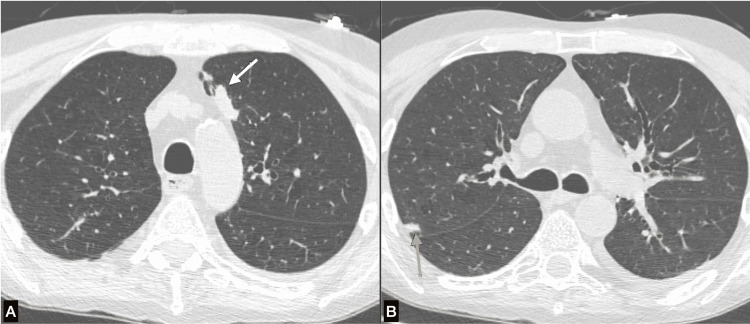
Axial CT image (A) shows a lobulated solid pulmonary nodule in the anterior segment of the left upper lobe adjacent to the mediastinal pleura (straight white arrow). Axial CT image (B) shows a small, slightly irregular-shaped solid pulmonary nodule in the posterior segment of the right upper lobe adjacent to the costal pleura (straight yellow arrow).

After 15 days of therapy, flucytosine was discontinued due to worsening pancytopenia, and the patient was started on fluconazole. Chest CT demonstrated bilateral pulmonary nodules concerning malignancy versus fungal infection (Figure 2).

**Figure 3:**
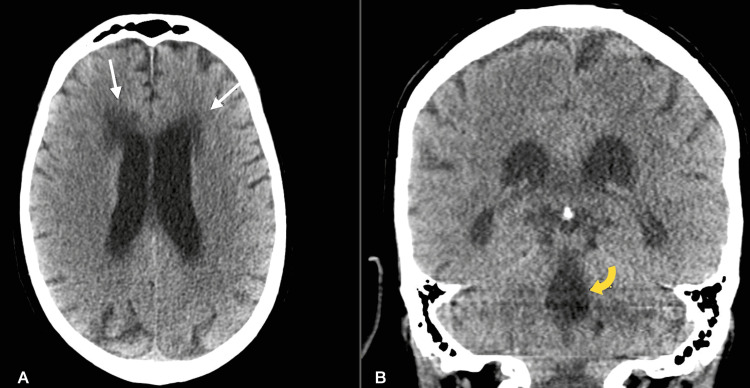
Axial CT image (A) shows new dilatation of ventricular system with white matter hypoattenuation adjacent to the ventricular system (straight white arrows), suggestive of acute hydrocephalus. Notice the dilated fourth ventricle (curved yellow arrow) on the coronal CT image (B), suggestive of communicating hydrocephalus.

Repeat CSF culture from prior LP demonstrated no fungal organisms, and amphotericin B was discontinued after 20 days of therapy. He remained afebrile, and his mental status improved. He was subsequently discharged on an eight-week maintenance course of oral fluconazole. One week following discharge, the patient was readmitted with worsening leukocytosis, fevers, and altered mental status. Head CT demonstrated new communicating hydrocephalus (Figure 3).

**Figure 4:**
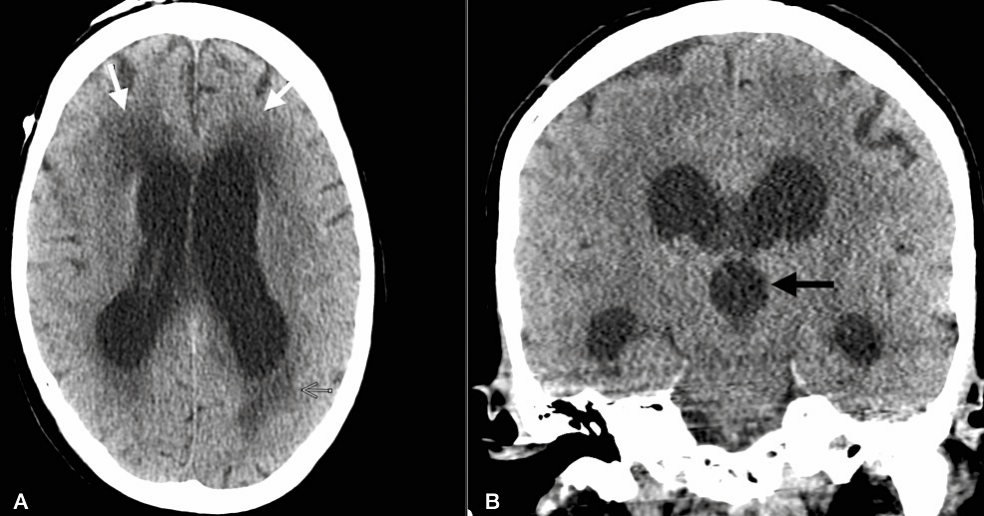
Axial CT image (A) shows marked enlargement of the ventricular system compared to previous CT with increased white matter hypoattenuation adjacent to the ventricular system (straight white arrows), suggestive of worsening hydrocephalus. Coronal CT image (B) shows dilated third ventricle (straight black arrow), which was not seen on previous CT, suggestive of worsening hydrocephalus.

CSF studies demonstrated persistently elevated cryptococcal antigen titer of 1:2560 with growth of *Cryptococcus* on CSF culture, although opening pressure remained low. Liposomal amphotericin B was reinitiated, and fluconazole was continued. His mental status progressively deteriorated to obtundation requiring transfer to the medical ICU. Repeat head CT showed interval worsening of hydrocephalus (Figure 4), and as a result, a ventriculoperitoneal shut was placed.

**Figure 5:**
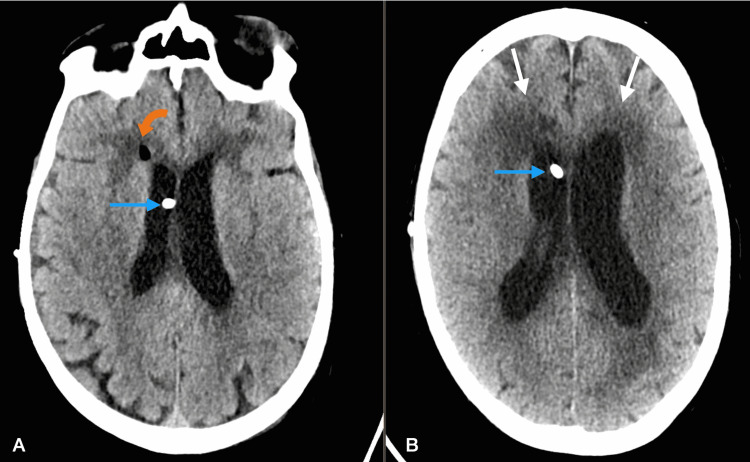
Axial CT image (A) obtained the next day shows almost normal size of ventricles, suggestive of improving hydrocephalus after placement of ventriculostomy catheter, which is seen coursing through the right lateral ventricle (straight blue arrow). A small focus of air (curved orange arrow) is seen, which is a normal postoperative finding. Follow-up axial CT image (B) obtained five days later shows dilatation of the ventricular system and worsened white matter hypoattenuation adjacent to the ventricular system (straight white arrows), suggestive of worsening hydrocephalus despite ventriculostomy catheter placement (straight blue arrow).

Despite the initial improvement of hydrocephalus on postoperative imaging, there was no evident improvement in the patient’s mental status. Over the following days, the patient remained markedly obtunded. Furthermore, follow-up head CT once again demonstrated progressively worsening hydrocephalus (Figure 5).

Ultimately, the patient’s care was transitioned to comfort measures only, and he expired on day 15 of admission, eight weeks after the initial detection of invasive cryptococcal infection.

## Discussion

Globally, cryptococcal CNS infections are generally associated with HIV infection [6]. Although in higher-income countries, it is increasingly becoming more prevalent in patients without HIV/AIDS, including those receiving immunosuppressive therapies, patients with connective tissue disorders, and transplant recipients [1,3,4,7,8].

Patients with underlying connective tissue disorders on immunosuppressive therapy have been associated with an increased risk for cryptococcosis. Our literature review indicates that the majority of reports of such groups focus on patients with systemic lupus erythematosus (SLE) or rheumatoid arthritis (RA), which are specific entities of connective tissue disease. In patients with rheumatoid arthritis (RA), both disease-related and iatrogenic immune dysfunction increases the risk of cryptococcosis, especially corticosteroid and TNF-α inhibitor use [9]. Given that TNF-α production is vital in the immune response against cryptococcal infection, TNF-α inhibitor use has been associated with cryptococcosis [10]. In one study of patients with rheumatoid arthritis with cryptococcosis, an increased risk was seen in patients with chronic kidney disease and those exposed to the anti-TNF antibody, adalimumab [11]. Given that our patient has mixed connective tissue disorder, we are unable to determine whether his condition is associated with any immune dysfunction, which may have contributed to his cryptococcal infection. While corticosteroid and TNF-α inhibitor use has been associated with cryptococcosis, there have been very few reported cases of cryptococcal infection in the setting of azathioprine therapy. Azathioprine, due to its ability to inhibit purine synthesis along with B and T lymphocytic cell function, can compromise the patients’ immune system and make them vulnerable to opportunistic infections, including cryptococcosis [12]. Therefore, in patients on immunosuppressive therapy who have invasive cryptococcal infection, one of the most important aspects of management is to reduce immunosuppressive therapy [5].

Cryptococcal CNS infections usually manifest in the form of meningitis, cryptococcomas, and/or hydrocephalus [13]. Complications of CNS infections include raised intracranial pressure and infarction, as seen in our patient. According to a study, infarction occurs in 26% of patients and is attributed to the ability of *Cryptococcus* species to invade brain endothelium, disrupting the blood-brain barrier and promoting panarteritis, thrombosis, and occlusion [14].

In terms of pharmacotherapy, amphotericin B and flucytosine are considered the first-line regimen for the induction phase [5]. However, some nonrandomized, weak evidence studies have shown some positive outcomes among HIV-negative patients with fluconazole monotherapy for cryptococcal meningitis [15,16]. The duration of therapy in cryptococcal meningitis depends on patient characteristics. Among organ transplant recipients, two weeks is considered adequate for induction, while non-HIV, non-transplant patients require 4-6 weeks for induction. Induction therapy is followed by eight weeks of consolidation phase usually with fluconazole, which is followed by 6-12 months of maintenance therapy of fluconazole [5].

Relapses of cryptococcosis have been reported to occur. A relapse is defined as a recurrence of symptoms or positive CSF cultures after the resolution of the primary infection. The rate of relapse is between 15% and 25% among non-HIV patients [5]. Most relapses have been attributed to inadequate dosing or duration of therapy during induction or maintenance. Given that our patient had negative CSF cultures following induction therapy, the recurrence of positive CSF study along with symptoms is indicative of relapse. We believe that the relapse may have occurred from the inadequate duration of induction therapy (20 days) as compared to 4-6 weeks of induction therapy recommended for non-HIV, non-transplant patients.

## Conclusions

Approximately 80% of cryptococcal infections occur in patients with HIV. Non-HIV, non-transplant recipient patients are the least numerous population groups affected by cryptococcal infections. Cryptococcal meningitis should be considered as a differential in non-HIV, non-transplant patients on azathioprine presenting with fever and worsening lethargy. In this patient group, 4-6 weeks of induction therapy is required to prevent relapse, whereas two weeks is considered adequate for induction in organ transplant recipients.
